# Association of Inherited Variation in Toll-Like Receptor Genes with Malignant Melanoma Susceptibility and Survival

**DOI:** 10.1371/journal.pone.0024370

**Published:** 2011-09-09

**Authors:** Andreas Gast, Justo Lorenzo Bermejo, Rainer Claus, Andreas Brandt, Marianne Weires, Alexander Weber, Christoph Plass, Antje Sucker, Kari Hemminki, Dirk Schadendorf, Rajiv Kumar

**Affiliations:** 1 Division of Molecular Genetic Epidemiology, German Cancer Research Center, Heidelberg, Germany; 2 Institute of Medical Biometry and Informatics, University of Heidelberg, Heidelberg, Germany; 3 Department of Epigenomics and Cancer Risk Factors, German Cancer Research Center, Heidelberg, Germany; 4 Junior Research Group Toll-like Receptors and Cancer, German Cancer Research Center, Heidelberg, Germany; 5 Department of Dermatology, University Hospital Essen, Essen, Germany; Charité-University Medicine Berlin, Germany

## Abstract

The family of Toll-like receptors (TLRs) is critical in linking innate and acquired immunity. Polymorphisms in the genes encoding *TLRs* have been associated with autoimmune diseases and cancer. We investigated the genetic variation of *TLR* genes and its potential impact on melanoma susceptibility and patient survival. The study included 763 cutaneous melanoma cases recruited in Germany and 736 matched controls that were genotyped for 47 single nucleotide polymorphisms (SNPs) in 8 *TLR* genes. The relationship between genotype, disease status and survival was investigated taking into account patient and tumor characteristics, and melanoma treatment. Analysis of 7 SNPs in *TLR2*, 7 SNPs in *TLR3* and 8 SNPs in *TLR4* showed statistically significant differences in distribution of inferred haplotypes between cases and controls. No individual polymorphism was associated with disease susceptibility except for the observed tendency for *TLR*2-rs3804099 (odds ratio OR  = 1.15, 95% CI 0.99–1.34, p = 0.07) and *TLR4*-rs2149356 (OR = 0.85, 95% CI 0.73–1.00, p = 0.06). Both polymorphisms were part of the haplotypes associated with risk modulation. An improved overall survival (Hazard ratio HR 0.53, 95% CI 0.32–0.88) and survival following metastasis (HR 0.55, 95% CI 0.34–0.91) were observed in carriers of the variant allele (D299G) of *TLR4*-rs4986790. In addition various TLR2, TLR4 and TLR5 haplotypes were associated with increased overall survival. Our results point to a novel association between *TLR* gene variants and haplotypes with melanoma survival. Our data suggest a role for the D299G polymorphism in the TLR4 gene in overall survival and a potential link with systemic treatment at stage IV of the disease. The polymorphic amino acid residue, located in the ectodomain of *TLR4*, can have functional consequences.

## Introduction

Malignant melanoma, with its propensity to metastasize and chemoresistance, remains a fatal neoplasm. Spontaneous regression, a phenomenon likely mediated by the immune system, is more common in melanoma than in most other cancers. The role of host immune system in melanoma is evidenced by the association of autoimmune conditions such as thyroiditis, vitiligo or the appearance of autoantibodies with an improved prognosis in patients treated with interleukin 2 and/or interferon α therapy [1], [2], [3], [4]. Variations in genes involved in critical pathways of the immune system might therefore influence the susceptibility to autoimmune diseases and subsequently melanoma susceptibility, progression and prognosis.

Toll-like receptors (TLR), the mammalian homologues of the Drosophila Toll protein, are perceived to be sensors for recognition of a number of invading pathogens.

TLR are critical in linking innate and acquired immunity and in serving as detectors of infectious pathogens and cancer debris [5], [6], [7]. TLRs belong to the family of pattern-recognition receptors (PRRs) which are expressed, among others, on antigen-presenting cells like dendritic cells or T-cells. After induction through pathogen-associated molecules, TLRs transduce signals via distinct intracellular pathways leading to the activation of transcription factors such as NFkB, interferon regulatory factors (IRFs) or AP-1. Finally these transcription receptors trigger inflammatory responses such as the release of inflammatory cytokines and type I interferons [7], [8], [9].

Polymorphisms in genes encoding TLR associated with infectious and non-infectious diseases, including autoimmunity and cancer, have been studied extensively [10], [11], [12], [13]. However, the variants in the genes have not been investigated for association with either melanoma susceptibility or disease outcome. In order to investigate the effect of polymorphisms in the *TLR* genes on melanoma susceptibility and survival, we genotyped 47 single nucleotide polymorphisms (SNP) in eight *TLR* genes in 763 cases from Germany and 736 ethnically matched controls.

## Results

The two multiplex reactions contained 373 replicates from different SNPs that showed a concordance rate over >99%. The rate of undetermined samples by the Sequenom procedure was between 6 and 33% of the individual SNPs. Genotype distributions of all polymorphisms in controls did not show statistically significant deviation from Hardy-Weinberg equilibrium.

Out of 47*TLR* SNPs analyzed by Sequenom mass spectrometry *TLR*1-rs3923647 and *TLR1*-rs5743613, *TLR*2-rs5743704 and -rs5743708, *TLR*4-rs11536869 and -rs11536897 and *TLR*6-rs5743815 had minor allele frequencies <0.05; no carrier of variant allele for *TLR*9-rs5743846 was detected. In total, the genotyping results of 47 SNPs represent 99 polymorphisms in the selected TLR genes.

### Case-control study

We did not observe any differences in the distribution of allele and genotype frequencies between melanoma cases and controls (Table S1, online only). The variant allele for *TLR*2-rs3804099 was associated with an increased risk (OR = 1.15, 95% CI 0.99–1.34, p = 0.07) and for *TLR*4-rs2149356 with a decreased risk (OR = 0.85, 95% CI 0.73–1.00, p = 0.06) of melanoma. However, for both, the association was not statistically significant.

### Haplotype analysis

Analysis of 7 SNPs in *TLR2*, 7 SNPs in *TLR3* and 8 SNPs in *TLR4* showed statistically significant differences in distribution of inferred haplotypes between cases and controls (Table 1). The haplotype A-C-C-C-C-G-T of the *TLR2* gene locus was associated with an increased risk compared to the consensus haplotype A-C-C-C-T-G-T (OR 1.31, 95%CI 1.03–1.65). This haplotype included the SNP that showed individually an association with increased risk. A haplotype for the *TLR3* gene (C-G-G-T-A-G-G) was associated with a decreased susceptibility to melanoma (OR 0.57, 95%CI 0.35–0.95) compared to the most frequent haplotype (C-G-A-C-C-C-A). The haplotype A-T-A-G-A-G-G-C of the *TLR4* gene locus associated with a decreased risk for melanoma compared to the most frequent haplotype A-T-C-A-A-G-G-T (OR 0.72, 95%CI 0.57–0.91). The haplotype includes the minor allele of *TLR2*-rs2149356 that associated individually with a protective effect. Haplotype analysis of the other *TLR*s did not identify associations with melanoma risk (data not shown).

**Table 1:** Distribution of the most common haplotypes within *TLR2*, *TLR3* and *TLR4* in German melanoma patients and German controls.

Gene	Haplotype	Cases (%)	Controls (%)	OR (95% CI)	*P*
*TLR2*	A-C-C-C-T-G-T	197 (12.9)	220 (15.0)	Reference	1.0
	T-C-A-C-T-G-T	491 (32.3)	496 (33.8)	1.11 (0.88–1.39)	
	A-C-C-C-C-G-T	457 (30.0)	391 (26.7)	**1.31 (1.03–1.65)**	
	T-T-C-C-C-G-T	140 (9.2)	141 (9.6)	1.11 (0.82–1.50)	
	A-C-C-C-C-G-C	94 (6.2)	85 (5.8)	1.23 (0.87–1.75)	
	A-C-C-A-T-G-T	48 (3.2)	51 (3.5)	1.05 (0.68–1.63)	
	T-C-A-C-T-A-T	38 (2.5)	33 (2.2)	1.29 (0.78–2.13)	
	T-C-C-C-T-G-T	29 (1.9)	24 (1.6)	1.35 (0.76–2.40)	
	T-T-C-C-T-G-T	12 (0.8)	11 (0.7)	1.22 (0.53–2.82)	
	other	16 (1.0)	15 (1.0)	−	
*TLR3*	C-G-A-C-C-C-A^3^	268 (17.6)	256 (17.5)	Reference	0.94
	C-A-G-C-C-G-G	240 (15.8)	211 (14.4)	1.09 (0.84–1.40)	
	C-G-G-T-A-C-G	205 (13.5)	187 (12.8)	1.05 (0.81–1.36)	
	C-G-G-C-A-C-G	153 (10.1)	176 (12)	0.83 (0.63–1.09)	
	T-G-G-C-C-C-G	215 (14.2)	175 (12)	1.17 (0.90–1.53)	
	C-G-G-C-C-C-A	123 (8.1)	128 (8.7)	0.92 (0.68–1.24)	
	C-A-G-C-C-C-G	91 (6.0)	77 (5.3)	1.13 (0.80–1.60)	
	C-G-A-C-C-C-G	71 (4.7)	75 (5.1)	0.90 (0.63–1.31)	
	C-G-G-T-A-G-G	27 (1.8)	45 (3.1)	**0.57 (0.35–0.95)**	
	C-G-G-C-C-C-G	44 (2.9)	33 (2.3)	1.27 (0.79–2.06)	
	C-G-G-C-C-G-G	16 (1.1)	20 (1.4)	0.76 (0.39–1.51)	
	T-G-G-C-C-C-A	13 (0.9)	14 (1.0)	0.89 (0.41–1.92)	
	C-G-G-C-C-G-A	16 (1.1)	13 (0.9)	1.18 (0.55–2.49)	
	other	37 (2.4)	53 (3.6)	−	
*TLR4*	A-T-C-A-A-G-G-T^3^	457 (30)	405 (27.5)	Reference	0.95
	A-C-C-G-A-G-G-T	371 (24.4)	357 (24.3)	0.92 (0.76–1.12)	
	A-T-A-G-A-G-G-C	192 (12.6)	237 (16.1)	**0.72 (0.57–0.91)**	
	A-C-C-G-A-C-G-T	209 (13.7)	215 (14.6)	0.86 (0.68–1.09)	
	A-T-A-G-G-G-G-C	85 (5.6)	77 (5.2)	0.98 (0.7–1.37)	
	G-T-A-G-A-G-G-T	43 (2.8)	52 (3.5)	0.73 (0.48–1.12)	
	A-T-A-G-A-G-G-T	60 (3.9)	40 (2.7)	1.33 (0.87–2.03)	
	A-T-A-G-A-G-A-C	50 (3.3)	39 (2.6)	1.14 (0.73–1.76)	
	A-C-A-G-A-G-G-T	8 (0.5)	10 (0.7)	0.71 (0.28–1.81)	
	other	46 (3.0)	40 (2.7)	−	

### Polymorphisms in *TLR* genes and survival

Differences in survival according to genotype were investigated based on 622 patients with melanoma of the skin with known primary at first diagnosis without detectable metastasis (stage I/II) (339 males and 283 females out of 763 patients in total).

**Figure 1:**
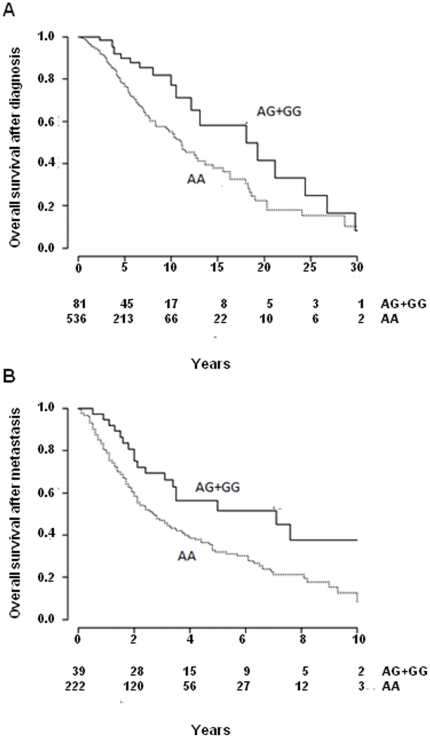
Overall survival (OS) and survival after metastasis (SFM) in German cutaneous melanoma patients according to the*TLR4*-rs4986790 (D299G) genotype. The Kaplan-Meier curves differentiate between carriers of the minor allele (AG+GG) and homozygote genotypes (AA) for (**A**) overall survival, log-rank *P* = 0.005 and (**B**) survival following the first metastasis, log-rank *P* = 0.01. Number of patients, both carriers and non-carriers, is given below the x-axis.

The OS from primary diagnosis was better in carriers of the minor allele (n = 81) of the *TLR4*-rs4986790 polymorphism than among non-carriers (n = 536). The median OS in carriers was 18.1 years (95% CI 12.1–24.4) compared to 11.0 years (95% CI 9.5–12.9) in non-carriers (log-rank *p* = 0.005, Figure 1A and Table 2). Cox regression adjusted for age, gender and Breslow thickness showed a decreased survival with a HR of 0.53, 95% CI 0.32–0.88, *p* = 0.01, Table 2) for carriers of the minor allele. Adjustment for any kind of therapy in stage IV showed no effect on this estimate (HR 0.53, 95% CI 0.32–0.87, *p* = 0.01, Table 2). Stratification according to therapy showed that the HR was 0.41 (95% CI 0.24–0.69, *p* = 0.001) among patients receiving any therapy, compared to HR 0.35 (95% CI 0.05–2.59, *p* = 0.30) for patients that did not receive any therapy. The statistical non-significance in the latter could be attributed to small patient numbers.

**Table 2:** *TLR4*-rs4986790 genotype and overall survival (OS), survival following the first metastasis (SFM) and metastasis free survival (MFS).

	Genotype	Individuals	Median survival in years (95% CI)	Uncensored events (%)	Hazard Ratio (95% CI)	*P*
**OS**	AA	536	11.0 (9.5–12.9)	161 (30.0)	Reference	
	AG	81	18.1 (12.1–24.4)	19 (23.5)	0.53 (0.32–0.88)	0.01
					0.53 (0.32–0.87)	0.01
**MFS**	AA	536	7.2 (5.5–8.3)	239 (44.6)	Reference	
	AG	81	10.6 (3.2–20.3)	39 (48.1)	0.81 (0.55–1.18)	0.27
**SFM**	AA	222	2.8 (2.1–3.5)	144 (64.9)	Reference	
	AG	39	7.1 (3.3–11.1)	19 (46.2)	0.55 (0.33–0.91)	0.02
					0.44 (0.27–0.73)	0.001

The *TLR4*-rs4986790 polymorphism was also associated with survival time following first metastasis (SFM). Carriers of the minor allele displayed a median SFM of 7.1 years (95% CI 3.3–11.1) compared to 2.8 years (95% CI 2.1–3.5) for non-carrier patients (log-rank *p* = 0.01, Figure 1B and Table 2). The effect was reflected in the Cox regression model with a HR of 0.55 (95% CI 0.33–0.91, *p* = 0.02) and adjustment for any therapy slightly decreased the estimate (HR 0.44, 95% CI 0.27–0.73, *p* = 0.001, Table 2). The stratification for therapy showed a statistically significant effect (HR 0.39, 95% CI 0.23–0.66, *p* = 0.001) only in patients that received therapy and not in patient that did not receive any therapy (HR 0.62, 95% CI 0.08–4.86, *p* = 0.65). The *TLR4*-rs4986790 polymorphism did not associate with time from primary diagnosis to first metastasis (Table 2).

Carriers of the variant allele of *TLR2*-rs3804100 (TC/CC) also showed a reduced OS (HR 0.55, 95% CI 0.29–1.05, p = 0.07). Adjustment for any therapy at any clinical stage did not show an effect (HR 0.52, 95% CI 0.27–1.01, p = 0.05). We also evaluated the possible interaction between polymorphisms *TLR4*-rs4986790 and *TLR2*-rs3804100 regarding OS. Patients with the commonest genotype (rs4986790 = AA, rs3804100 = TT) showed the poorest prognosis. Among 13 patients with rs4986790 = AG and rs3804100 = TC/CC, no death was observed ten years after diagnosis. The survival heterogeneity among the four categories of individuals resulted in a probability value from the log-rank test of 0.01, which increased to 0.03 after inclusion of age, gender and Breslow thickness in a subsequent Cox regression. Other investigated polymorphisms did not associate with OS, SFM or MFS.

### *Association of TLR* haplotypes with overall survival

Several haplotypes inferred based on polymorphisms in *TLR2*, *TLR4* and *TLR5* showed association with OS (Table 3). In the *TLR2* gene, a minor haplotype A-C-C-C-C-G-C (5.5%) was significantly associated with an increased survival compared to the consensus haplotype A-C-C-C-T-G-T (HR 0.46, 95% CI 0.22 - 0.95). Another haplotype in *TLR4*, A-T-A-G-A-G-G-T, associated with OS (HR 1.77, 95% CI 1.02–3.08) in comparison to the most frequent haplotype. A frequent haplotype of *TLR4* (A-T-A-G-A-G-G-C, 13.9%) also associated with increased OS (HR 1.4, 95% CI 0.95–2.06). Out of the 5 major TLR5 haplotypes, one associated with enhanced OS (T-T-G-C, HR 1.6, 95% CI 1.05–2.43). The remaining *TLR* haplotypes did not show significant association with OS (data not shown).

**Table 3:** Overall survival according to the commonest *TLR2*, *TLR4* and *TLR5* haplotypes.

Gene	Haplotype	Frequency (%)	Uncensored events (%)	HR (95% CI)	*P*
*TLR2*	A-C-C-C-T-G-T	121 (11.2)	37 (3.4)	Reference	0.11
	T-C-A-C-T-G-T	367 (33.9)	105 (9.7)	0.86 (0.60–1.24)	
	A-C-C-C-C-G-T	345 (31.9)	100 (9.2)	0.84 (0.58–1.24)	
	T-T-C-C-C-G-T	104 (9.6)	32 (3.0)	1.07 (0.67–1.73)	
	A-C-C-C-C-G-C	59 (5.5)	9 (0.8)	**0.46 (0.22–0.95)**	
	A-C-C-A-T-G-T	33 (3.0)	8 (0.7)	0.62 (0.28–1.36)	
	T-C-A-C-T-A-T	20 (1.8)	1 (0.1)	0.24 (0.03–1.78)	
	T-C-C-C-T-G-T	20 (1.8)	4 (0.4)	0.58 (0.21–1.65)	
	other	13 (1.2)	6 (0.6)	−	
*TLR4*	A-T-C-A-A-G-G-T^3^	323 (29.9)	82 (7.6)	Reference	0.12
	A-C-C-G-A-G-G-T	268 (24.8)	85 (7.9)	1.09 (0.77–1.53)	
	A-T-A-G-A-G-G-C	150 (13.9)	47 (4.3)	1.40 (0.95–2.06)	
	A-C-C-G-A-C-G-T	139 (12.9)	35 (3.2)	1.01 (0.67–1.50)	
	A-T-A-G-G-G-G-C	68 (6.3)	17 (1.6)	0.60 (0.35–1.04)	
	A-T-A-G-A-G-G-T	40 (3.7)	16 (1.5)	**1.77 (1.02–3.08)**	
	A-T-A-G-A-G-A-C	32 (3.0)	5 (0.5)	0.44 (0.18–1.10)	
	G-T-A-G-A-G-G-T	29 (2.7)	9 (0.8)	1.73 (0.83–3.60)	
	other	33 (3.1)	6 (0.6)	−	
*TLR5*	C-T-G-C^3^	241 (22.3)	61 (5.7)	Reference	0.32
	C-T-C-C	395 (36.6)	108 (10.0)	1.18 (0.85–1.63)	
	C-G-G-C	287 (26.6)	86 (8.0)	1.33 (0.91–1.93)	
	T-T-G-C	96 (8.9)	35 (3.2)	**1.60 (1.05–2.43)**	
	C-T-C-T	53 (4.9)	12 (1.1)	0.96 (0.51–1.83)	
	other	8 (0.7)	1 (0.1)	−	

### *TLR* Haplotype associations with survival time following first metastasis

The *TLR2* haplotype T-T-C-C-C-G-T (9.6%) was associated with an increased SFM compared to the common haplotype (HR 1.54 95% CI 1.04–2.28). Other significant associations with decreased SFM were seen for *TLR4*- A-T-A-G-A-G-A-C and *TLR4*-G-T-A-G-A-G-G-T (HR 0.36, 95% CI 0.14–0.90 and HR 2.41, 95% CI 1.10–5.24), which were both rare (3.1% and 2.7%, respectively). Two frequent haplotypes A-C-C-G-A-G-G-T and A-T-A-G-A-G-G-C (26.2% and 14.1%, respectively) in this gene were associated with decreased SFM (HR 1.36, 95% CI 0.97–1.92 and HR 1.47, 95% CI 0.99–2.19, respectively). No other of the *TLR* haplotypes showed association with SFM.

### *TLR* Haplotype associations with metastasis free survival (MFS) after diagnosis of primary tumor

Patients that harbored the *TLR4* haplotype A-T-A-G-A-G-G-T were at an increased risk for MFS compared to those carrying the most frequent haplotype A-T-C-A-A-G-G-T (HR 2.02, 95% CI 1.28–3.17). Significant association with MFS was also seen for one of the most frequent haplotypes (C-G-G-C, frequency 26.5%) of the *TLR5* gene when compared to the consensus haplotype C-T-G-C (HR 1.39, 95% CI 1.03–1.88). Other associations of *TLR* haplotypes with MFS were not statistically significant.

## Discussion

TLRs, the mammalian homologues of Drosophila toll protein, play a role as agents of innate and adaptive immunity in various diseases. We assumed that genetic polymorphisms in various *TLRs* were functional and influenced melanoma susceptibility and disease outcome. Our data showed that melanoma patients carrying the variant allele for the rs4986790 polymorphism in the *TLR4* gene were associated with a prolonged OS. The increase in survival was confined to the period after the detection of a first metastasis, which implied that the effect of the polymorphism might possibly modulated dependent on therapy or alternatively by biological processes associated with melanoma cell migration and/or invasion. The patients in stage IV melanoma in most instances received a chemotherapy containing DTIC/temozolomide and interferons, which is known for long time not having any clinical effect on overall survival of the entire metastatic melanoma population [14], [15].

One can speculate that the prolonged survival after first metastasis, which clinically is in most cases regionally spread, is mediated by more efficacious tumor control at the lymph node level and by interaction with the immune system.

*TLR4*-rs4986790 polymorphism results in a change of amino acid D299G and it co-segregates with a T399I exchange, which are both located in the ectodomain of TLR4 [16]. The ectodomain of TLR4 is responsible for detecting of lipopolysaccharides from Gram negative bacteria in cooperation with an accessory molecule, myeloid differentiation factor 2 (MD-2). MD-2 forms a 1∶1 complex with lipopolysaccharides, which is detected by TLR4 [16]. Interestingly, the amino acid residues 299 and 399 of TLR4 are not located either in the recognition surface of TLR4 for the MD-2-lipopolysaccharide complex or on the surface required for receptor dimerization. Nevertheless, both polymorphisms reduce responsiveness to lipopolysaccharides as shown in tissue culture systems with re-constituted TLR4 mutants [17]. Apart from bacterial lipopolysaccharides, TLR4 was shown to be activated by host high mobility group box 1 protein (HMGB-1) released as a result of chemo- or radiotherapy, and potential tissue damage [18]. In that study, on French breast cancer patients, the 299G form of TLR4 showed reduced binding to HMGB1 and a correlation with faster relapse after radiotherapy [18]. It is interesting to speculate whether HMGB1, released as a consequence of tissue necrosis or therapy, results in genotype dependent differential activation of TLR4, which contributes to the observed differences in survival. Further patient-based and functional studies, will be required to confirm the correlation between polymorphisms and survival. It is also worth noting that colon cancer patients carrying the minor allele of *TLR4*-rs4986790 exhibited worse progression free survival and OS after treatment with chemotherapy [19]. On the other hand, a comprehensive Swedish study on 20 *TLR* signaling pathway genes found significant association between two polymorphisms with prostate cancer mortality that did not include *TLR4*-rs4986790 [20]. Thus, even if modulated HMGB-1 signaling might be due to the effects of *TLR4*-rs4986790, the outcome in terms of OS is difficult to predict as the TLR ligation can have pleiotropic effects on cellular differentiation and growth.

With the exception of two polymorphisms, *TLR*2-rs3804099 and *TLR*4-rs2149356, none other of the 47 variants in eight *TLR* genes included in this study showed association with risk of melanoma in comparison to the healthy controls. The variant alleles of the two polymorphisms showed a tendency towards association with susceptibility but not strong enough to rule out a chance finding. Nevertheless, previous reports have shown associations of the rs3804099 polymorphism in the *TLR*2 gene with sepsis in preterm infants [21]. It was also associated with reversal reaction in leprosy and tuberculosis in Ethiopian and Vietnamese populations [22], [23]. On the other hand no association of this SNP could be found with normal tension glaucoma in a Japanese population or asthma in a German and a mixed German/Austrian population or type I diabetes mellitus in a Basque population [24], [25], [26], [27]. Since the polymorphism is synonymous (N199N) and not linked with any other variant in the gene, the association with various diseases could be due to linkage with other SNPs outside the *TLR2* gene region. The most commonly discussed polymorphisms in *TLR2*, the R677W and R753Q, have been shown to be associated, amongst other diseases, with leprosy, tuberculosis, staphylococcal infections, coronary restenosis and sepsis [11], [12]. In our study the R677W could not be included due to immeasurable genotype, probably caused by a pseudogene located upstream of the true gene [28]. The R753Q variant was rather rare and did not show any association with melanoma susceptibility.

The variant allele of *TLR4*-rs2149356, exhibited association with decreased risk of melanoma that was not statistically significant. However, the haplotype containing the same variant allele was associated with statistically significant decreased risk. *TLR4*-rs2149356 is located in intron 2 of the gene and is linked with four other polymorphisms at the locus. One of which is located in intron 1 (rs1927911) and three (rs10116253, rs2737190, rs1927914) in the 5′end of the gene. Therefore it is possible that SNPs in the 5′ end could possibly effect transcriptional regulation. In previous studies variants in the *TLR*4 gene have been shown to be associated with infectious and non-infectious diseases but rather inconsistently [10], [29], [30], [31].

In conclusion, on the role of variants in eight *TLR* genes in malignant melanoma show an association of one variant in the *TLR4* gene with the disease survival. Though we did not find any clear association for any polymorphism with the disease susceptibility, for two SNPs we did find a tendency for such an association that also extended into a frequent haplotype in *TLR4* gene.

## Materials and Methods

### Patients and controls

763 patients of German origin (418 male, 345 female) with cutaneous melanoma were recruited at the Skin Cancer Unit at the Mannheim University Hospital, Germany, according to eligibility criteria that included histologically confirmed melanoma of the skin; AJCC stage 0 (in situ melanoma, 10), I (358), II (253), III (112) and IV (11) disease and 19 tumors had an unknown stage (Table 4). Median and mean age of the melanoma cases at diagnosis was 55 and 54.1 years, respectively. Mean Breslow thickness was 2.14 mm (95% confidence interval 1.97 to 2.31 mm).

**Table 4:** Characteristics of the German patients included in the study.

	*All patients*	*Patients with AJCC stage 0, I or II at first diagnosis (FD)*
*No.*	763	622
*Gender*		
Male	418 (54.8%)	341 (38.8%)
Female	345 (45.2%)	281 (45.2%)
*Age at first diagnosis(FD)*		
Median	55	54
Mean	54.1	54.9
*Breslow thickness (mm)*		
Mean (95% CI)	2.14 (1.97–2.31)	1.81 (1.66–1.96)
*Ulceration of primary tumor*		
Yes	130 (17.0)%)	92 (14.8%)
No	185 (24.3%)	159 (25.6%)
unknown	448 (58.7)	371 (59.7%)
*AJCC stage at FD*		
0	10 (1.3%)	10 (1.6%)
I	396 (51.9%)	396 (63.7%)
II	216 (28.3%)	216 (34.7%)
III	111 (14.6%)	−
IV	11 (1.4%)	−
unknown	19 (2.5%)	−
*Metastasized during follow-up*	237 (31.1%)	237 (38.1%)%)
*Deceased during follow-up*	170 (22.3%)	127 (20.4%)
*Therapy*		
No therapy	408 (53.5%)	364 (58.5%)
Any therapy	353 (46.3%)	258 (41.5%)
Chemotherapy	28 (3.7%)	17 (2.7%)
Immunotherapy	129 (16.9)	98 (15.8%)
All other therapy combinations	199 (26.1%)	143 (23.0%)

Blood samples from case subjects were taken during the first appointment at the Skin Cancer Unit. Controls included 736 healthy individuals (368 male and 368 female) recruited at the Institute of Transfusion Medicine and Immunology in Mannheim, Germany. Median and mean age of the controls were 61 and 60.3 years, respectively. The ethical approval for the study was granted by Ethics Commission of the Faculty for Clinical Medicine of Ruprecht-Karls-Universität, Heidelberg and written informed consent was obtained from all study participants. DNA was extracted from blood samples using Qiagen mini-preparation kits.

### Selection of SNPs

Selection of SNPs in eight human *TLR* genes was done by inclusion of known non-synonymous SNPs, those in regulatory regions or reported by other investigators. Additionally tagging SNPs for each gene region were selected from HapMap data using the criteria, a) exclusion of individuals with >50% missing genotypes, b) pair-wise r^2^>0.8 for each SNP pair and c) minor allele frequencies >5%. Of the eight *TLR* genes, three were located in a gene cluster on chromosome 4p14 (*TLR6-TLR1-TLR10*). For tagging analysis, the region from rs10024216 to rs6531668 (69.5 kb) was selected (Figure S1, online only). *TLR2* and *TLR3* are located on chromosome arm 4q; these two genes were about 32 Mb apart from each other and were not linked. *TLR4* is on chromosome 9q23; TLR5 on 1q41 and TLR9 on 3p21. In total we selected 47 SNPs that were informative for 99 polymorphisms in 8 genes. The selected SNPs were grouped into 2 multiplex assays (W1 and W2) for the Sequenom mass array platform (Sequenom, San Diego, CA, USA, Table S2 and S3, online only). In addition, the *TLR*4-rs4986790 (D299G) SNP was selected for genotyping using an allelic discrimination method.

### Genotyping

Genotyping was carried out with the Sequenom method. In two multiplex PCR (with the 25 or 23 primer pairs, respectively) DNA fragments with SNPs were amplified using 10 ng DNA as template in 5 µl volume. Genotype analysis was performed by the Sequenom TYPER 4.0 software. Validation of genotype data for SNPs that showed statistically significant association (*TLR*2-rs3804099 and *TLR*4-rs2149356) was done by allelic discrimination method and DNA sequencing.

### Statistical analysis

All statistical calculations were carried out using SAS version 9.2 (SAS Institute, Cary, NC, USA). Odds ratios (OR) with the corresponding 95% confidence intervals (95% CI) for assessment of the association between risk and genotype were based on logistic regression adjusted for age, gender and Breslow thickness. The haplotype procedure of SAS genetics was used to estimate haplotype frequencies in cases and controls separately, and to infer possible haplotype combinations for each individual. The evidence of association between genotype/haplotype and melanoma risk were summarized by probability values.

Overall survival (OS) was defined as the time (in years) from date of first diagnosis until death or last patient contact. Metastasis free survival (MFS) was the time from diagnosis of primary melanoma until the first metastasis (either regional or distant). Survival following first metastasis (SFM) was defined as the time from first metastasis to death or last patient contact. Alive patients at the latest visit/contact were considered censored. Univariate survival curves were based on the Kaplan-Meier method and statistical significance was quantified by log-rank tests. Genotype-specific survival differences were adjusted for age, gender and Breslow thickness, based on a proportional hazard regression (Cox) model for each of the polymorphisms. Ulceration status was not included in the multivariate analysis due to unavailability of complete data. Subsequently, the analysis of the potential association between genotype and survival was adjusted for therapy administered in stage IV. We considered the start time and duration of therapy, treating therapy as a time-dependent covariate. The possible interaction between therapy and genotype was explored by including the interaction in the Cox regression and, additionally, by stratification of patients into therapy groups.

Association of haplotypes with survival time was carried out by Cox regression. Thereby, we assumed that individuals carry the most likely combination of possible haplotypes and that the effects of haplotypes interact multiplicatively. Hazard ratios (HR) were calculated taking the consensus haplotype as reference. If the frequency of the consensus haplotype was low, the most common haplotype was taken as reference.

## Supporting Information

Figure S1 **Linkage disequilibrium (LD) plot of the 69-kb** ***TLR6-TLR1-TLR10*** **gene cluster on chromosome 4p14.** The plot was drawn using Haploview software 4.2 and based on HapMap homepage. It shows r^2−^values, the higher r^2^, the darker the box. LD blocks are depicted as triangles. Positions of the *TLR* genes and SNPs are shown in the upper part of the figure.
(DOC)

Table S1 **Genotype distributions of TLR SNPs in malignant melanoma of the skin in patients and controls.**
(DOC)

Table S2 **Toll-like receptor genes and polymorphisms.**
(DOC)

Table S3 **List of iplex primer and masses.**
(XLS)
