# Supplementary figures and images for: Association of Inherited Variation in Toll-Like Receptor Genes with Malignant Melanoma Susceptibility and Survival
Source: PLoS One. 2011 Sep 9;6(9):e24370. doi: 10.1371/journal.pone.0024370 (PMC3170315; doi:10.1371/journal.pone.0024370)

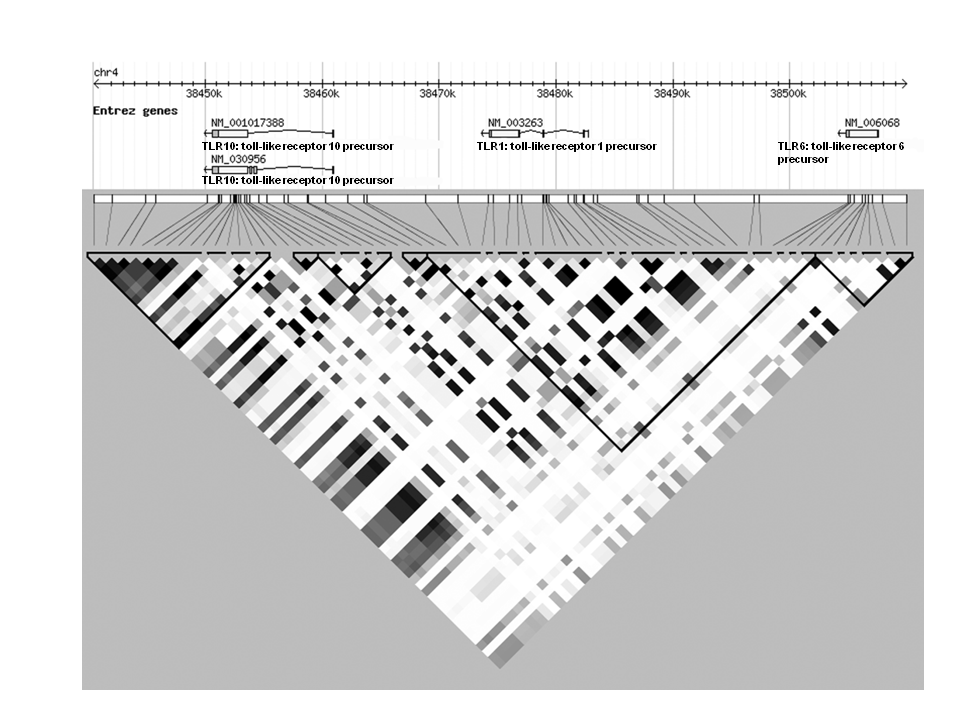

Supplement: Figure S1 — Linkage disequilibrium (LD) plot of the 69-kb TLR6-TLR1-TLR10 gene cluster on chromosome 4p14. The plot was drawn using Haploview software 4.2 and based on HapMap homepage. It shows r2−values, the higher r2, the darker the box. LD blocks are depicted as triangles. Positions of the TLR genes and SNPs are shown in the upper part of the figure. (DOC) [file pone.0024370.s001.doc]
