# Supplementary material for: Association of Inherited Variation in Toll-Like Receptor Genes with Malignant Melanoma Susceptibility and Survival
Source: PLoS One. 2011 Sep 9;6(9):e24370. doi: 10.1371/journal.pone.0024370 (PMC3170315; doi:10.1371/journal.pone.0024370)
Supplement: Table S1 — Genotype distributions of TLR SNPs in malignant melanoma of the skin in patients and controls. (DOC) [file pone.0024370.s002.doc]

**Table S1. Genotype distributions of TLR SNPs in malignant melanoma of the skin in patients and controls from Germany.**

| **Gene** | **Chr. Pos.[[1]](#endnote-2)** | **SNP ID** | **Loc.[[2]](#endnote-3) , AA change** | **Allele** | **Cases (%)** | **Controls (%)** | **OR (95% CI)** | ***P*** |
| --- | --- | --- | --- | --- | --- | --- | --- | --- |
| *TLR6* | 4p14 | rs5743788 | 5´ near | CC | 209 (29.4) | 171 (26.0) | Reference | 0.13[[3]](#endnote-4) |
|  |  |  |  | CG | 348 (48.9) | 356 (54.2) | 0.81 (0.62−1.05) |  |
|  |  |  |  | GG | 154 (21.7) | 130 (19.8) | 1.03 (0.75−1.42) |  |
|  |  |  |  | C | 766 (53.9) | 698 (53.1) | Reference | 0.99[[4]](#endnote-5) |
|  |  |  |  | G | 656 (46.1) | 616 (46.9) | 1.0 (0.86−1.17) |  |
|  |  |  |  |  |  |  |  |  |
|  |  | rs5743789 | 5´ near | TT | 436 (63.5) | 375 (61.9) | Reference | 0.63 |
|  |  |  |  | TA | 225 (32.8) | 204 (33.7) | 0.96 (0.75−1.22) |  |
|  |  |  |  | AA | 26 (3.8) | 27 (4.5) | 0.76 (0.43−1.35) |  |
|  |  |  |  | T | 1097 (79.8) | 954 (78.7) | Reference | 0.42 |
|  |  |  |  | A | 277 (20.2) | 258 (21.3) | 0.92 (0.75−1.12) |  |
|  |  |  |  |  |  |  |  |  |
|  |  | rs5743806 | 5´ near | TT | 316 (45.3) | 276 (45.2) | Reference | 0.20 |
|  |  |  |  | TC | 298 (42.7) | 282 (46.2) | 0.91 (0.72−1.16) |  |
|  |  |  |  | CC | 84 (12.0) | 53 (8.7) | 1.31 (0.88−1.95) |  |
|  |  |  |  | T | 930 (66.6) | 834 (68.3) | Reference | 0.56 |
|  |  |  |  | C | 466 (33.4) | 388 (31.8) | 1.05 (0.89−1.25) |  |
|  |  |  |  |  |  |  |  |  |
|  |  | rs3821985 | exon 1, T361T | CC | 302 (42.4) | 282 (42.2) | Reference | 0.45 |
|  |  |  |  | CG | 311 (43.6) | 313 (46.9) | 0.95 (0.75−1.20) |  |
|  |  |  |  | GG | 100 (14.0) | 73 (10.9) | 1.20 (0.84−1.71) |  |
|  |  |  |  | C | 915 (64.2) | 877 (65.6) | Reference | 0.57 |
|  |  |  |  | G | 511 (35.8) | 459 (34.4) | 1.05 (0.89−1.23) |  |
|  |  |  |  |  |  |  |  |  |
|  |  | rs5743815 | exon 1, V427A | TT | 704 (97.6) | 650 (97.2) | Reference | 0.77 |
|  |  |  |  | TC | 16 (2.2) | 19 (2.8) | 0.78 (0.39−1.56) |  |
|  |  |  |  | CC | 1 (0.1) | 0 (0.0) | nd[[5]](#endnote-6) |  |
|  |  |  |  | T | 1424 (98.8) | 1319 (98.6) | Reference | 0.66 |
|  |  |  |  | C | 18 (1.3) | 19 (1.4) | 0.86 (0.44−1.68) |  |
|  |  |  |  |  |  |  |  |  |
| *TLR1* | 4p14 | rs4833103 | 5´ near | AA | 186 (26.7) | 157 (25.8) | Reference | 0.65 |
|  |  |  |  | AC | 374 (53.6) | 316 (52.0) | 0.95 (0.72−1.24) |  |
|  |  |  |  | CC | 137 (19.7) | 135 (22.2) | 0.86 (0.62−1.19) |  |
|  |  |  |  | A | 746 (53.5) | 630 (51.8) | Reference | 0.38 |
|  |  |  |  | C | 648 (46.5) | 586 (48.2) | 0.93 (0.79−1.09) |  |
|  |  |  |  |  |  |  |  |  |
|  |  | rs5743566 | 5´ UTR | CC | 466 (66.7) | 394 (64.2) | Reference | 0.50 |
|  |  |  |  | CG | 205 (29.4) | 200 (32.6) | 0.88 (0.69−1.12) |  |
|  |  |  |  | GG | 27 (3.9) | 20 (3.3) | 1.13 (0.61−2.10) |  |
|  |  |  |  | C | 1137 (81.4) | 988 (80.5) | Reference | 0.57 |
|  |  |  |  | G | 259 (18.6) | 240 (19.5) | 0.94 (0.77−1.15) |  |
|  |  |  |  |  |  |  |  |  |
|  |  | rs5743594 | intron 2 | CC | 477 (65.5) | 431 (64.2) | Reference | 0.45 |
|  |  |  |  | CT | 224 (30.8) | 218 (32.5) | 0.88 (0.69−1.11) |  |
|  |  |  |  | TT | 27 (3.7) | 22 (3.3) | 1.18 (0.65−2.16) |  |
|  |  |  |  | C | 1178 (80.9) | 1080 (80.5) | Reference | 0.62 |
|  |  |  |  | T | 278 (19.1) | 262 (19.5) | 0.95 (0.78−1.16) |  |
|  |  |  |  |  |  |  |  |  |
|  |  | rs5743595 | intron 2 | TT | 477 (67.0) | 431 (66.2) | Reference | 0.59 |
|  |  |  |  | TC | 200 (28.1) | 196 (30.1) | 0.94 (0.74−1.21) |  |
|  |  |  |  | CC | 35 (4.9) | 24 (3.7) | 1.27 (0.73−2.21) |  |
|  |  |  |  | T | 1154 (81.0) | 1058 (81.3) | Reference | 0.84 |
|  |  |  |  | C | 270 (19.0) | 244 (18.7) | 1.02 (0.84−1.24) |  |
|  |  |  |  |  |  |  |  |  |
|  |  | rs5743611 | exon 4, R80T | GG | 396 (97.5) | 393 (78.8) | Reference | 0.74 |
|  |  |  |  | GC | 96 (19.3) | 103 (20.6) | 0.89 (0.64−1.25) |  |
|  |  |  |  | CC | 6 (1.2) | 3 (0.6) | 1.33 (0.32−5.60) |  |
|  |  |  |  | G | 888 (89.2) | 889 (89.1) | Reference | 0.66 |
|  |  |  |  | C | 108 (10.8) | 109 (10.9) | 0.94 (0.69−1.26) |  |
|  |  |  |  |  |  |  |  |  |
|  |  | rs4833095 | exon 4, N248S | TT | 363 (59.5) | 374 (58.6) | Reference | 0.86 |
|  |  |  |  | TC | 204 (33.4) | 217 (34.0) | 0.93 (0.73−1.20) |  |
|  |  |  |  | CC | 43 (7.1) | 47 (7.4) | 0.97 (0.61−1.53) |  |
|  |  |  |  | T | 930 (76.2) | 965 (75.6) | Reference | 0.65 |
|  |  |  |  | C | 290 (23.8) | 311 (24.4) | 0.96 (0.79−1.16) |  |
|  |  |  |  |  |  |  |  |  |
|  |  | rs3923647 | exon 4, H305L | AA | 666 (94.9) | 593 (94.6) | Reference | 0.96 |
|  |  |  |  | AT | 35 (5.0) | 34 (5.4) | 0.93 (0.57−1.54) |  |
|  |  |  |  | TT | 1 (0.1) | 0 (0.0) | nd |  |
|  |  |  |  | A | 1367 (97.4) | 1220 (97.3) | Reference | 0.97 |
|  |  |  |  | T | 37 (2.6) | 34 (2.7) | 0.99 (0.61−1.61) |  |
|  |  |  |  |  |  |  |  |  |
|  |  | rs5743613 | exon 4, P315L | CC | 724 (99.5) | 672 (99) | Reference | nd |
|  |  |  |  | CT | 0 (0.0) | 5 (0.7) | nd |  |
|  |  |  |  | TT | 4 (0.5) | 2 (0.3) | nd |  |
|  |  |  |  | C | 1448 (99.5) | 1349 (99.3) | Reference | 0.94 |
|  |  |  |  | T | 8 (0.5) | 9 (0.7) | 0.96 (0.36−2.60) |  |
|  |  |  |  |  |  |  |  |  |
| *TLR10* | 4p14 | rs10856839 | 5´ UTR | AA | 492 (69.3) | 474 (71.0) | Reference | 0.81 |
|  |  |  |  | AC | 197 (27.7) | 176 (26.3) | 1.09 (0.85−1.39) |  |
|  |  |  |  | CC | 21 (3.0) | 18 (2.7) | 1.07 (0.55−2.08) |  |
|  |  |  |  | A | 1181 (83.2) | 1124 (84.1) | Reference | 0.54 |
|  |  |  |  | C | 239 (16.8) | 212 (15.9) | 1.07 (0.87−1.37) |  |
|  |  |  |  |  |  |  |  |  |
|  |  | rs11466652 | exon 2, K303K | AA | 528 (72.7) | 490 (76.3) | Reference | 0.08 |
|  |  |  |  | AG | 184 (25.3) | 134 (20.9) | 1.27 (0.98−1.65) |  |
|  |  |  |  | GG | 14 (1.9) | 18 (2.8) | 0.63 (0.30−1.31) |  |
|  |  |  |  | A | 1240 (85.4) | 1114 (86.8) | Reference | 0.45 |
|  |  |  |  | G | 212 (14.6) | 170 (13.2) | 1.09 (0.87−1.37) |  |
|  |  |  |  |  |  |  |  |  |
|  |  | rs11096956 | exon 2, P344P | GG | 477 (63.4) | 440 (62.4) | Reference | 0.36 |
|  |  |  |  | GT | 239 (31.7) | 239 (33.9) | 0.94 (0.75−1.18) |  |
|  |  |  |  | TT | 37 (4.9) | 26 (3.7) | 1.40 (0.82−2.39) |  |
|  |  |  |  | G | 1193 (79.2) | 1119 (79.4) | Reference | 0.73 |
|  |  |  |  | T | 313 (20.8) | 291 (20.6) | 1.03 (0.86−1.24) |  |
|  |  |  |  |  |  |  |  |  |
|  |  | rs11096955 | exon 4, I369L | AA | 299 (43.1) | 238 (39.4) | Reference | 0.13 |
|  |  |  |  | AC | 290 (41.9) | 278 (46.0) | 0.79 (0.61−1.00) |  |
|  |  |  |  | CC | 104 (15.0) | 88 (14.6) | 0.98 (0.69−1.34) |  |
|  |  |  |  | A | 888 (64.1) | 754 (62.4) | Reference | 0.41 |
|  |  |  |  | C | 498 (35.9) | 454 (37.6) | 0.93 (0.79−1.10) |  |
|  |  |  |  |  |  |  |  |  |
|  |  | rs4129009 | exon 4, V775L | AA | 468 (68.8) | 392 (64.4) | Reference | 0.53 |
|  |  |  |  | AG | 195 (27.3) | 191 (31.4) | 0.88 (0.70−1.13) |  |
|  |  |  |  | GG | 27 (3.9) | 26 (4.3) | 0.82 (0.46−1.46) |  |
|  |  |  |  | A | 1131 (82.0) | 975 (80.1) | Reference | 0.26 |
|  |  |  |  | G | 249 (18.0) | 243 (20) | 0.89 (0.73−1.09) |  |
|  |  |  |  |  |  |  |  |  |
|  |  | rs9715841 | exon 4, 3´UTR | TT | 456 (64.2) | 402 (61.4) | Reference | 0.59 |
|  |  |  |  | TC | 219 (30.9) | 223 (34.1) | 0.90 (0.71−1.14) |  |
|  |  |  |  | CC | 35 (4.9) | 30 (4.6) | 1.09 (0.65−1.85) |  |
|  |  |  |  | T | 1131 (79.6) | 1027 (78.4) | Reference | 0.66 |
|  |  |  |  | C | 289 (20.4) | 283 (21.6) | 0.96 (0.79−1.16) |  |
|  |  |  |  |  |  |  |  |  |
| *TLR2* | 4q32 | rs4696480 | intron 1 | AA | 192 (27.6) | 156 (25.4) | Reference | 0.63 |
|  |  |  |  | AT | 347 (49.8) | 315 (51.2) | 0.92 (0.70−1.20) |  |
|  |  |  |  | TT | 158 (22.7) | 144 (23.4) | 0.85 (0.62−1.18) |  |
|  |  |  |  | A | 731 (52.4) | 627 (51.0) | Reference | 0.34 |
|  |  |  |  | T | 663 (47.6) | 603 (49.0) | 0.93 (0.80−1.09) |  |
|  |  |  |  |  |  |  |  |  |
|  |  | rs1816702 | intron 2, | CC | 563 (81.0) | 482 (79.8) | Reference | 0.24 |
|  |  |  |  | CT | 123 (17.7) | 120 (19.9) | 0.86 (0.64−1.15) |  |
|  |  |  |  | TT | 9 (1.3) | 2 (0.3) | 2.87 (0.59−14.0) |  |
|  |  |  |  | C | 1249 (89.9) | 1084 (89.7) | Reference | 0.67 |
|  |  |  |  | T | 141 (10.1) | 124 (10.3) | 0.94 (0.73−1.23) |  |
|  |  |  |  |  |  |  |  |  |
|  |  | rs11938228 | intron 2 | CC | 292 (41.8) | 246 (40.2) | Reference | 0.71 |
|  |  |  |  | CA | 318 (45.6) | 282 (46.1) | 0.93 (0.73−1.18) |  |
|  |  |  |  | AA | 88 (12.6) | 84 (13.7) | 0.87 (0.61−1.25) |  |
|  |  |  |  | C | 902 (64.6) | 774 (63.2) | Reference | 0.41 |
|  |  |  |  | A | 494 (35.4) | 450 (36.8) | 0.93 (0.79−1.10) |  |
|  |  |  |  |  |  |  |  |  |
|  |  | rs5743704 | exon 3, P631H | CC | 648 (93.4) | 579 (92.3) | Reference | 0.27 |
|  |  |  |  | CA | 46 (6.6) | 48 (7.7) | 0.78 (0.51−1.21) |  |
|  |  |  |  | AA | 0 | 0 | nd |  |
|  |  |  |  | C | 1342 (96.7) | 1206 (96.2) | Reference | 0.28 |
|  |  |  |  | A | 46 (3.3) | 48 (3.8) | 0.79 (0.52−1.21) |  |
|  |  |  |  |  |  |  |  |  |
|  |  | rs3804099[[6]](#endnote-7) | exon 3, N199N | TT | 220 (28.9) | 246 (33.7) | Reference | 0.17 |
|  |  |  |  | TC | 381 (50.1) | 345 (47.3) | 1.21 (0.95−1.54) |  |
|  |  |  |  | CC | 160 (21.0) | 138 (18.9) | 1.30 (0.96−1.76) |  |
|  |  |  |  | T | 821 (53.9) | 837 (57.4) | Reference | 0.07 |
|  |  |  |  | C | 701 (46.1) | 621 (42.6) | 1.15 (0.99−1.34) |  |
|  |  |  |  |  |  |  |  |  |
|  |  | rs5743708 | exon 3, R753Q | GG | 661 (95.0) | 585 (95) | Reference | 0.95 |
|  |  |  |  | GA | 35 (5.0) | 30 (4.9) | 0.92 (0.55−1.54) |  |
|  |  |  |  | AA | 0 (0.0) | 1 (0.2) | nd |  |
|  |  |  |  | G | 1357 (97.5) | 1200 (97.4) | Reference | 0.57 |
|  |  |  |  | A | 35 (2.5) | 32 (2.6) | 0.86 (0.52−1.43) |  |
|  |  |  |  |  |  |  |  |  |
|  |  | rs3804100 | exon 3, S450S | TT | 617 (88.1) | 589 (88.3) | Reference | 0.92 |
|  |  |  |  | TC | 81 (11.6) | 75 (11.2) | 1.01 (0.72−1.43) |  |
|  |  |  |  | CC | 2 (0.3) | 3 (0.5) | 0.69 (0.11−4.15) |  |
|  |  |  |  | T | 1315 (93.9) | 1253 (93.9) | Reference | 0.93 |
|  |  |  |  | C | 85 (6.1 | 81 (6.1) | 0.99 (0.71−1.36) |  |
|  |  |  |  |  |  |  |  |  |
| *TLR3* | 4q35 | rs11730143 | intron 1 | CC | 355 (70.0) | 419 (72.2) | Reference | 0.30 |
|  |  |  |  | CT | 137 (27.0) | 151 (26) | 1.14 (0.86−1.52) |  |
|  |  |  |  | TT | 15 (3.0) | 10 (1.7) | 1.79 (0.77−4.18) |  |
|  |  |  |  | C | 847 (83.5) | 989 (85.3) | Reference | 0.16 |
|  |  |  |  | T | 167 (16.5) | 171 (14.7) | 1.19 (0.93−1.52) |  |
|  |  |  |  |  |  |  |  |  |
|  |  | rs7657186 | intron 1 | GG | 438 (61.3) | 423 (63.5) | Reference | 0.44 |
|  |  |  |  | GA | 239 (33.4) | 219 (32.9) | 1.01 (0.80−1.28) |  |
|  |  |  |  | AA | 38 (5.3) | 24 (3.6) | 1.44 (0.82−2.50) |  |
|  |  |  |  | G | 1115 (78.0) | 1065 (80.0) | Reference | 0.40 |
|  |  |  |  | A | 315 (22.0) | 267 (20.0) | 1.09 (0.90−1.31) |  |
|  |  |  |  |  |  |  |  |  |
|  |  | rs13126816 | intron 1 | GG | 412 (58.8) | 362 (58.5) | Reference | 0.95 |
|  |  |  |  | GA | 256 (36.5) | 226 (36.5) | 0.96 (0.76−1.22) |  |
|  |  |  |  | AA | 33 (4.7) | 31 (5.0) | 1.01 (0.60−1.70) |  |
|  |  |  |  | G | 1080 (76.9) | 950 (76.7) | Reference | 0.84 |
|  |  |  |  | A | 322 (23.1) | 288 (23.3) | 0.98 (0.81−1.18) |  |
|  |  |  |  |  |  |  |  |  |
|  |  | rs5743312 | intron 3 | CC | 500 (69.8) | 453 (69.0) | Reference | 0.51 |
|  |  |  |  | CT | 200 (27.9) | 193 (29.4) | 0.93 (0.73−1.19) |  |
|  |  |  |  | TT | 16 (2.2) | 11 (1.7) | 1.47 (0.66−3.30) |  |
|  |  |  |  | C | 1200 (83.8 | 1099 (83.6) | Reference | 0.96 |
|  |  |  |  | T | 232 (16.2) | 215 (16.4) | 0.99 (0.81−1.23) |  |
|  |  |  |  |  |  |  |  |  |
|  |  | rs7668666 | intron 3 | CC | 376 (52.3) | 331 (49.7) | Reference | 0.45 |
|  |  |  |  | CA | 297 (41.3) | 280 (42) | 0.95 (0.76−1.20) |  |
|  |  |  |  | AA | 46 (6.4) | 55 (8.3) | 0.76 (0.49−1.17) |  |
|  |  |  |  | C | 1049 (72.9) | 942 (70.7) | Reference | 0.29 |
|  |  |  |  | A | 389 (27.1) | 390 (29.3) | 0.91 (0.77−1.08) |  |
|  |  |  |  |  |  |  |  |  |
|  |  | rs3775292 | intron 3 | CC | 464 (63.6) | 413 (61.8) | Reference | 0.71 |
|  |  |  |  | CG | 233 (31.9) | 228 (34.1) | 0.92 (0.72−1.16) |  |
|  |  |  |  | GG | 33 (4.5) | 27 (4.0) | 1.07 (0.62−1.85) |  |
|  |  |  |  | C | 1161 (79.5) | 1054 (78.9) | Reference | 0.68 |
|  |  |  |  | G | 299 (20.5) | 282 (21.1) | 0.96 (0.80−1.16) |  |
|  |  |  |  |  |  |  |  |  |
|  |  | rs3775291 | exon 4, L412F | GG | 379 (51.8) | 332 (49.7) | Reference | 0.44 |
|  |  |  |  | GA | 291 (39.7) | 284 (42.5) | 0.88 (0.70−1.11) |  |
|  |  |  |  | AA | 62 (8.5) | 52 (7.8) | 1.09 (0.72−1.64) |  |
|  |  |  |  | G | 1049 (71.7) | 948 (71) | Reference | 0.73 |
|  |  |  |  | A | 415 (28.3) | 388 (29) | 0.97 (0.82−1.15) |  |
|  |  |  |  |  |  |  |  |  |
| *TLR4* | 9q33.1 | rs11536869 | intron 1 | AA | 683 (93.8) | 626 (92.9) | Reference | 0.90 |
|  |  |  |  | AG | 47 (6.2) | 47 (7.0) | 0.9 (0.58−1.40) |  |
|  |  |  |  | GG | 0 (0.0) | 1 (0.2) | nd |  |
|  |  |  |  | A | 1411 (96.9) | 1299 (96.4) | Reference | 0.53 |
|  |  |  |  | G | 45 (3.1) | 49 (3.6) | 0.87 (0.57−1.34) |  |
|  |  |  |  |  |  |  |  |  |
|  |  | rs12377632 | intron 2 | TT | 267 (37.5) | 226 (34.2) | Reference | 0.43 |
|  |  |  |  | TC | 336 (47.1) | 327 (49.6) | 0.86 (0.67−1.09) |  |
|  |  |  |  | CC | 110 (15.4) | 107 (16.2) | 0.87 (0.63−1.21) |  |
|  |  |  |  | T | 870 (61.0) | 779 (59) | Reference | 0.29 |
|  |  |  |  | C | 556 (39.0) | 541 (41) | 0.92 (0.78−1.08) |  |
|  |  |  |  |  |  |  |  |  |
|  |  | rs2149356[[7]](#endnote-8)8 | intron 2 | CC | 371 (49.2) | 331 (45.5) | Reference | 0.13 |
|  |  |  |  | CA | 323 (42.8) | 322 (44.3) | 0.89 (0.71−1.11) |  |
|  |  |  |  | AA | 60 (8.0) | 74 (10.2) | 0.68 (0.46−1.00) |  |
|  |  |  |  | C | 1065 (70.6) | 984 (67.7) | Reference | 0.06 |
|  |  |  |  | A | 443 (29.4) | 470 (32.3) | 0.85 (0.73−1.00) |  |
|  |  |  |  |  |  |  |  |  |
|  |  | rs5030728 | intron 2 | GG | 325 (47.2) | 341 (52.1) | Reference | 0.18 |
|  |  |  |  | GA | 304 (44.1) | 261 (39.9) | 1.23 (0.97−1.55) |  |
|  |  |  |  | AA | 60 (8.7) | 52 (8.0) | 1.27 (0.84−1.92) |  |
|  |  |  |  | G | 954 (69.2) | 943 (72.1) | Reference | 0.08 |
|  |  |  |  | A | 424 (30.8) | 365 (27.9) | 1.17 (0.98−1.38) |  |
|  |  |  |  |  |  |  |  |  |
|  |  |  |  |  |  |  |  |  |
|  |  | rs4986790 | exon 3, D299G | AA | 665 (88.0) | 659 (89.7) | Reference | 0.61 |
|  |  |  |  | AG | 91 (12.0) | 73 (9.9) | 1.19 (0.85−1.66) |  |
|  |  |  |  | GG | 0 (0.0) | 3 (0.4) | nd |  |
|  |  |  |  | A | 1330 (93.6) | 1318 (94.4) | Reference | 0.57 |
|  |  |  |  | G | 91 (6.4) | 79 (5.7) | 1.10 (0.80−1.51) |  |
|  |  |  |  |  |  |  |  |  |
|  |  | rs11536889 | 3´ UTR | GG | 532 (73.9) | 471 (71.3) | Reference | 0.46 |
|  |  |  |  | GC | 167 (23.2) | 172 (26.0) | 0.85 (0.66−1.10) |  |
|  |  |  |  | CC | 21 (2.9) | 18 (2.7) | 0.9 (0.46−1.75) |  |
|  |  |  |  | G | 1231 (85.5) | 1114 (84.3) | Reference | 0.25 |
|  |  |  |  | C | 209 (14.5) | 208 (15.7) | 0.88 (0.71−1.09) |  |
|  |  |  |  |  |  |  |  |  |
|  |  | rs11536897 | 3´ near | GG | 669 (93.3) | 638 (94.7) | Reference | 0.65 |
|  |  |  |  | GA | 47 (6.6) | 35 (5.2) | 1.25 (0.78−2.02) |  |
|  |  |  |  | AA | 1 (0.1) | 1 (0.2) | nd |  |
|  |  |  |  | G | 1385 (96.6) | 1311 (97.3) | Reference | 0.37 |
|  |  |  |  | A | 49 (3.4) | 37 (2.7) | 1.24 (0.78−1.95) |  |
|  |  |  |  |  |  |  |  |  |
|  |  | rs1554973 | 3´ near | TT | 400 (58.0) | 333 (55.1) | Reference | 0.14 |
|  |  |  |  | TC | 261 (37.9) | 237 (39.2) | 0.90 (0.71−1.15) |  |
|  |  |  |  | CC | 28 (4.1) | 34 (5.6) | 0.58 (0.34−1.01) |  |
|  |  |  |  | T | 1061 (77.0) | 903 (74.8) | Reference | 0.09 |
|  |  |  |  | C | 317 (23) | 305 (25.3) | 0.85 (0.71−1.03) |  |
|  |  |  |  |  |  |  |  |  |
| *TLR5* | 1q41 | rs2241096 | intron 3 | CC | 577 (79.8) | 532 (79.3) | Reference | 0.46 |
|  |  |  |  | CT | 143 (19.8) | 134 (20.0) | 0.94 (0.71−1.23) |  |
|  |  |  |  | TT | 3 (0.4) | 5 (0.8) | 0.40 (0.09−1.82) |  |
|  |  |  |  | C | 1297 (89.7) | 1198 (89.3) | Reference | 0.42 |
|  |  |  |  | T | 149 (10.3) | 144 (10.7) | 0.90 (0.70−1.16) |  |
|  |  |  |  |  |  |  |  |  |
|  |  | rs2241097 | intron 5 | TT | 375 (54.2) | 330 (54.4) | Reference | 0.90 |
|  |  |  |  | TG | 268 (38.8) | 240 (39.5) | 1.04 (0.82−1.31) |  |
|  |  |  |  | GG | 48 (7.0) | 37 (6.1) | 1.11 (0.69−1.77) |  |
|  |  |  |  | T | 1018 (73.7) | 900 (74.1) | Reference | 0.66 |
|  |  |  |  | G | 364 (26.3) | 314 (25.9) | 1.04 (0.87−1.25) |  |
|  |  |  |  |  |  |  |  |  |
|  |  | rs851192 | intron 5 | GG | 251 (35.7) | 209 (31.7) | Reference | 0.27 |
|  |  |  |  | GC | 317 (45.0) | 323 (48.9) | 0.81 (0.64−1.04) |  |
|  |  |  |  | CC | 136 (19.3) | 128 (19.4) | 0.88 (0.64−1.21) |  |
|  |  |  |  | G | 819 (58.2) | 741 (56.1) | Reference | 0.28 |
|  |  |  |  | C | 589 (41.8) | 579 (43.9) | 0.92 (0.78−1.07) |  |
|  |  |  |  |  |  |  |  |  |
|  |  | rs5744168 | exon 6, R392X | CC | 638 (89.1) | 591 (89.1) | Reference | 1.00 |
|  |  |  |  | CT | 78 (10.9) | 71 (10.7) | 1.01 (0.71−1.44) |  |
|  |  |  |  | TT | 0 (0.0) | 1 (0.2) | nd |  |
|  |  |  |  | C | 1354 (94.6) | 1253 (94.5) | Reference |  |
|  |  |  |  | T | 78 (5.5) | 73 (5.5) | 0.99 (0.70−1.39) | 0.91 |
|  |  |  |  |  |  |  |  |  |
| *TLR9* | 3p21.1 | rs5743836 | 5´ near | TT | 509 (72.0) | 469 (76.4) | Reference | 0.26 |
|  |  |  |  | TC | 172 (24.7) | 135 (22.0) | 1.24 (0.95−1.63) |  |
|  |  |  |  | CC | 16 (2.3) | 10 (1.6) | 1.25 (0.54−2.89) |  |
|  |  |  |  | T | 1190 (85.4) | 1073 (87.4) | Reference | 0.12 |
|  |  |  |  | C | 204 (14.6) | 155 (12.6) | 1.21 (0.96−1.53) |  |
|  |  |  |  |  |  |  |  |  |
|  |  | rs187084 | 5´ near | TT | 238 (34.2) | 215 (35.2) | Reference | 0.42 |
|  |  |  |  | TC | 334 (47.9) | 278 (45.5) | 1.09 (0.85−1.41) |  |
|  |  |  |  | CC | 125 (17.9) | 118 (19.3) | 0.89 (0.64−1.23) |  |
|  |  |  |  | T | 810 (58.1) | 708 (57.9) | Reference | 0.64 |
|  |  |  |  | C | 584 (41.9) | 514 (42.1) | 0.96 (0.82−1.13) |  |

1. Chr. Pos. = chromosomal position [↑](#endnote-ref-2)
2. Loc. = localisation, AA = amino acid [↑](#endnote-ref-3)
3. Global probability value from three-genotype model. [↑](#endnote-ref-4)
4. *P*-value for allelic effects. [↑](#endnote-ref-5)
5. nd = not determined [↑](#endnote-ref-6)
6. results from allelic discrimination [↑](#endnote-ref-7)
7. ^8^ [↑](#endnote-ref-8)
