# Supplementary material for: Association of Inherited Variation in Toll-Like Receptor Genes with Malignant Melanoma Susceptibility and Survival
Source: PLoS One. 2011 Sep 9;6(9):e24370. doi: 10.1371/journal.pone.0024370 (PMC3170315; doi:10.1371/journal.pone.0024370)
Supplement: Table S2 — Toll-like receptor genes and polymorphisms. (DOC) [file pone.0024370.s003.doc]

Table S2. Toll-like receptor genes and polymorphisms.

| **Gene** | **[[1]](#endnote-2)Chromosomal Position** | **[[2]](#endnote-3)Locus , AA change** | **SNP ID** | **Polymorphism** |
| --- | --- | --- | --- | --- |
| ***TLR1*** | 4p14 | 5´ near | rs4833103 | A>C |
|  |  | 5´ UTR | rs5743566 | C>G |
|  |  | intron 2 | rs5743594 | C>T |
|  |  | intron 2 | rs5743595 | T>C |
|  |  | exon 4, R80T | rs5743611 | G>C |
|  |  | exon 4, N248S | rs4833095 | T>C |
|  |  | exon 4, H305L | rs3923647 | A>T |
|  |  | exon 4, P315L | rs5743613 | C>T |
|  |  |  |  |  |
| ***TLR2*** | 4q32 | intron 1 | rs4696480 | A>T |
|  |  | intron 2, | rs1816702 | C>T |
|  |  | intron 2 | rs11938228 | C>A |
|  |  | exon 3, P631H | rs5743704 | C>A |
|  |  | exon 3, N199N | [[3]](#endnote-4)rs3804099 | T>C |
|  |  | exon 3, R753Q | rs5743708 | G>A |
|  |  | exon 3, S450S | rs3804100 | T>C |
|  |  |  |  |  |
| ***TLR3*** | 4q35 | intron 1 | rs11730143 | C>T |
|  |  | intron 1 | rs7657186 | G>A |
|  |  | intron 1 | rs13126816 | G>A |
|  |  | intron 3 | rs5743312 | C>T |
|  |  | intron 3 | rs7668666 | C>A |
|  |  | intron 3 | rs3775292 | C>G |
|  |  | exon 4, L412F | rs3775291 | G>A |
|  |  |  |  |  |
| ***TLR4*** | 9q33.1 | intron 1 | rs11536869 | A>G |
|  |  | intron 2 | rs12377632 | T>C |
|  |  | intron 2 | [[4]](#endnote-5)rs21493568 | C>A |
|  |  | intron 2 | rs5030728 | G>A |
|  |  | exon 3, D299G | rs4986790 | A>G |
|  |  | 3´ UTR | rs11536889 | G>C |
|  |  | 3´ near | rs11536897 | G>A |
|  |  | 3´ near | rs1554973 | T>C |
|  |  |  |  |  |
| ***TLR5*** | 1q41 | intron 3 | rs2241096 | C>T |
|  |  | intron 5 | rs2241097 | T>G |
|  |  | intron 5 | rs851192 | G>C |
|  |  | exon 6, R392X | rs5744168 | C>T |
|  |  |  |  |  |
| ***TLR6*** | 4p14 | 5´ near | rs5743788 | C>G |
|  |  | 5´ near | rs5743789 | T>A |
|  |  | 5´ near | rs5743806 | T>C |
|  |  | exon 1, T361T | rs3821985 | C>G |
|  |  | exon 1, V427A | rs5743815 | T>C |
|  |  |  |  |  |
| ***TLR9*** | 3p21.1 | 5´ near | rs5743836 | T>C |
|  |  | 5´ near | rs187084 | T>C |
|  |  | exon 2, A882T | rs5743836 | G>A |
|  |  |  |  |  |
| ***TLR10*** | 4p14 | 5´ UTR | rs10856839 | A>C |
|  |  | exon 2, K303K | rs11466652 | A>G |
|  |  | exon 2, P344P | rs11096956 | G>T |
|  |  | exon 4, I369L | rs11096955 | A>C |
|  |  | exon 4, V775L | rs4129009 | A>G |
|  |  | exon 4, 3´UTR | rs9715841 | T>C |

1. C [↑](#endnote-ref-2)
2. L [↑](#endnote-ref-3)
3. r [↑](#endnote-ref-4)
4. r [↑](#endnote-ref-5)
